# Validation of subpixel target detection and linear spectral unmixing techniques on short-wave infrared hyperspectral images of collagen phantoms

**DOI:** 10.1117/1.JBO.30.2.023518

**Published:** 2025-02-25

**Authors:** Hsian-Min Chen, Hsin-Che Wang, Chiu-Chin Sung, Yu-Ting Hsu, Yi-Jing Sheen

**Affiliations:** a Taichung Veterans General Hospital, Center for Quantitative Imaging in Medicine (CQUIM), Department of Medical Research, Taichung, Taiwan; b National Yang Ming Chiao Tung University, Institute of Biomedical Informatics, Taipei, Taiwan; c Taichung Veterans General Hospital, Division of Endocrinology and Metabolism, Department of Internal Medicine, Taichung, Taiwan; d National Yang Ming Chiao Tung University, School of Medicine, Department of Medicine, Taipei, Taiwan; e National Chung Hsing University, Department of Post-Baccalaureate Medicine, Taichung, Taiwan

**Keywords:** short-wave infrared hyperspectral imaging, subpixel target detection, linear spectral unmixing, collagen phantoms, constrained energy minimization, fully constrained least squares

## Abstract

**Significance:**

We used three-dimensionally printed experimental molds and designed lard (lipid)–collagen mixed phantoms to simulate biological tissues to verify the practicality and accuracy of short-wave infrared (SWIR) hyperspectral imaging (HSI; 900 to 1700 nm), subpixel target detection (STD), and linear spectral unmixing (LSU). We provide a foundation for future development, validation, and reproducibility of hyperspectral image-processing techniques.

**Aim:**

We aim to verify the use of SWIR HSI in bionic tissue phantoms. Second, we focus on the accuracy of STD and spectral unmixing techniques in hyperspectral image processing. Finally, the penetration ability of the technology and its applications at various depths and concentrations are explored.

**Approach:**

All experiments were conducted using an SWIR (900 to 1700 nm) HSI sensor. Collagen phantoms of different thicknesses were created to test the penetration abilities. Lard (lipid) was embedded at different depths in the phantoms for STD, whereas LSU was performed on phantoms with varying collagen concentrations. The methods used included constrained energy minimization to detect the lard target and fully constrained least squares (FCLS) to estimate the abundance of collagen phantoms.

**Results:**

SWIR HSI effectively penetrated the collagen phantoms. Specifically, STD techniques can accurately detect the presence of lard (lipids) at depths of 7 to 20 mm in the collagen phantoms. Even at a depth of 68 mm, the detection accuracy was 0.907. Moreover, in the LSU analysis, the FCLS method accurately estimated the abundance of collagen phantoms at different concentrations, with a correlation coefficient of 0.9917, indicating high accuracy across different concentrations.

**Conclusions:**

This study demonstrated that SWIR HSI is highly accurate for deep target detection and LSU. This technology has great potential for use in future noninvasive biomedical diagnostic models. Collagen phantoms are valuable tools for validating HSI algorithms and provide a solid foundation for clinical applications.

## 1 Introduction

Water and lipids constitute >60% of the human body. Studies have shown that these components are closely associated with cell function.^1^ Other studies have suggested that changes in the concentration and spatial distribution of these components are markers of many diseases, including cardiovascular diseases,^2^ diabetes,^3^^,^^4^ and cancer.^5^ Another study indicated that aging and diabetes can cause protein glycation, leading to dysfunction of collagen-containing tissues.^6^ Changes in collagen structure and function can affect the development of pathological changes in the skin, blood vessels, and nerves, leading to complications, an increased risk of disability, and life-threatening conditions. These studies showed that water, lipids, and collagen play crucial roles in human health.

Hyperspectral imaging (HSI) is widely used in clinical and biomedical research, particularly in tissue analysis and disease diagnosis. This technique is particularly effective in the short-wave infrared (SWIR) range, where key biological molecules, such as water, lipids, and collagen, exhibit distinctive absorption characteristics.^7^^–^^9^ The water absorption peaks at 970 and 1180 nm correspond to the vibrational overtones of the O–H bond, whereas the peak at 1430 nm arises from the first overtone of the O–H stretching. Lipids exhibit absorption peaks at 920, 1210, 1730, and 1760 nm, which were attributed to overtones and combinations of –e H bond vibrational modes. Collagen, a critical structural protein, shows peaks at 1200, 1500, and 1725 nm owing to overtones and combination bands of C–H and ${\mathrm{CH}}_{2}$ stretching.^7^^,^^10^^,^^11^ These molecular-specific absorption features in the SWIR range enable precise tissue characterization, highlighting the suitability of SWIR HSI for biomedical applications. Recent studies have demonstrated the practical applications of SWIR imaging in both preclinical and clinical settings. For example, SWIR mesopatterned imaging has been used to map water and lipid concentrations in mouse models of inflammation and edema, as well as in human superficial blood vessels, enabling noninvasive monitoring of lipid levels and spatial distribution.^12^^,^^13^ These findings underscore the potential of SWIR HSI for advancing tissue characterization and monitoring physiological processes *in vivo*.

For specific tissue components of interest, we can measure the absorption and scattering coefficients of *ex vivo* tissues using an integrating sphere system^14^^,^^15^ or measure the reflected or scattered spectra from the surface of *in vivo* tissues using a single-point fiber-optic system.^11^^,^^16^ Although the integrated sphere system can measure the diffuse reflectance and transmittance spectra of the samples and provide accurate absorption and scattering coefficients, it is limited to *ex vivo* samples and cannot measure live or dynamic tissues. The use of a single-point fiber-optic probe system solves this issue by enabling effective reflectance or scattering spectral measurements of any biological tissue. However, this method is limited by the fiber probe size and optical path length, making it unsuitable for large-scale spectral tissue analyses.

In recent years, advancements in SWIR HSI sensor technology and reduced costs have made commercial HSI more accessible. HSI can cover larger sample areas and provide rich spatial and spectral information, overcoming the limitations of traditional single-point fiber-optic hyperspectral techniques for wide-area measurements. This study also provides valuable insights into the optical properties and physiological status of biological tissues for clinical applications. For instance, hyperspectral imaging can be used to estimate tissue absorption and scattering coefficients, assess melanin concentration^17^ and distribution,^18^ measure oxygen saturation levels,^19^ and differentiate disease severity based on spectral differences.^20^^,^^21^

Saager et al.^17^ measured the optical properties of the forearms and upper inner arms of 12 subjects in the 450- to 1000-nm wavelength range to assess the melanin concentration and distribution depth. Kono and Yamada^18^ developed a reflection spatial profile measurement system covering the 450- to 800-nm and 950- to 1600-nm wavelength ranges to estimate the optical properties of the skin and explore the effects of individual differences, age, sex, and body regions on the optical properties in visible and near-infrared wavelengths. Teaw et al.^19^ proposed the noninvasive measurement of oxyhemoglobin and deoxyhemoglobin concentrations in patients with systemic sclerosis using HSI to assess the severity and activity of Raynaud’s phenomenon. Chen et al.^20^ and Sheen et al.^21^ used SWIR HSI to study patients with scleroderma and diabetic foot neuropathy and used spectral difference analysis to distinguish disease severity. Their results demonstrate the feasibility of HSI technology for clinical diagnosis. Merdasa et al.^22^ combined HSI and spectral unmixing techniques to measure oxygen saturation during forehead flap reconstruction. Spectral unmixing enables real-time mapping of clinically relevant oxygen saturation values and offers a spatially resolved oxygen monitoring technique during surgery.

The use of visible (400 to 1000 nm) or SWIR (900 to 1700 nm) HSI for human skin tissue analysis is limited by the ability of light to penetrate the tissues. Previous studies^23^ have reported a penetration depth of 1 to 15 mm in the skin tissue depending on the light wavelength (200 to 1600 nm). One study^23^ using SWIR HSI of chicken breast tissues of varying thicknesses found that the penetration depth of the SWIR light reached at least 12 mm.

From an anatomical perspective, hyperspectral images obtained at such depths contain mixed spectral features from various skin tissues such as blood vessels, water, collagen, and lipids. To analyze the spectral characteristics of the skin tissue using spectral unmixing methods, we first assumed the number of endmembers (components) present in the tissue. It is typically difficult to precisely determine this answer when analyzing live skin tissue using HSI. Therefore, obtaining a standard reference from live tissue hyperspectral images is challenging if we want to conduct repeated studies on different hyperspectral image-processing methods to verify the algorithmic accuracy.

The main purpose of this study is to verify the accuracy and practicality of two commonly used methods in hyperspectral image processing, subpixel detection, and linear spectral unmixing (LSU), by designing collagen phantoms that mimic biological tissues. First, we confirmed the penetration ability of SWIR HSI into the designed collagen phantoms. This ensures that the HSI can capture spectral signals from targets at different depths, allowing us to test the capability of the subpixel target detection (STD) method-constrained energy minimization (CEM) to detect targets at varying depths.

We also designed collagen phantoms with four different collagen concentrations (i.e., 2%, 3%, 4%, and 5%) and captured hyperspectral images using SWIR HSI. We then applied the LSU–fully constrained least squares (LSU-FCLS) method to estimate the abundance of the collagen phantoms and verified its accuracy in estimating the target content.

This study verified the potential and limitations of SWIR HSI and image-processing techniques in biomedical applications by analyzing the hyperspectral images of phantoms of different depths, mixtures, and concentrations. This study provides scientific evidence for its use in clinical diagnosis.

## 2 Materials and Methods

### 2.1 SWIR HSI and Imaging Platform

**Fig. 1:**
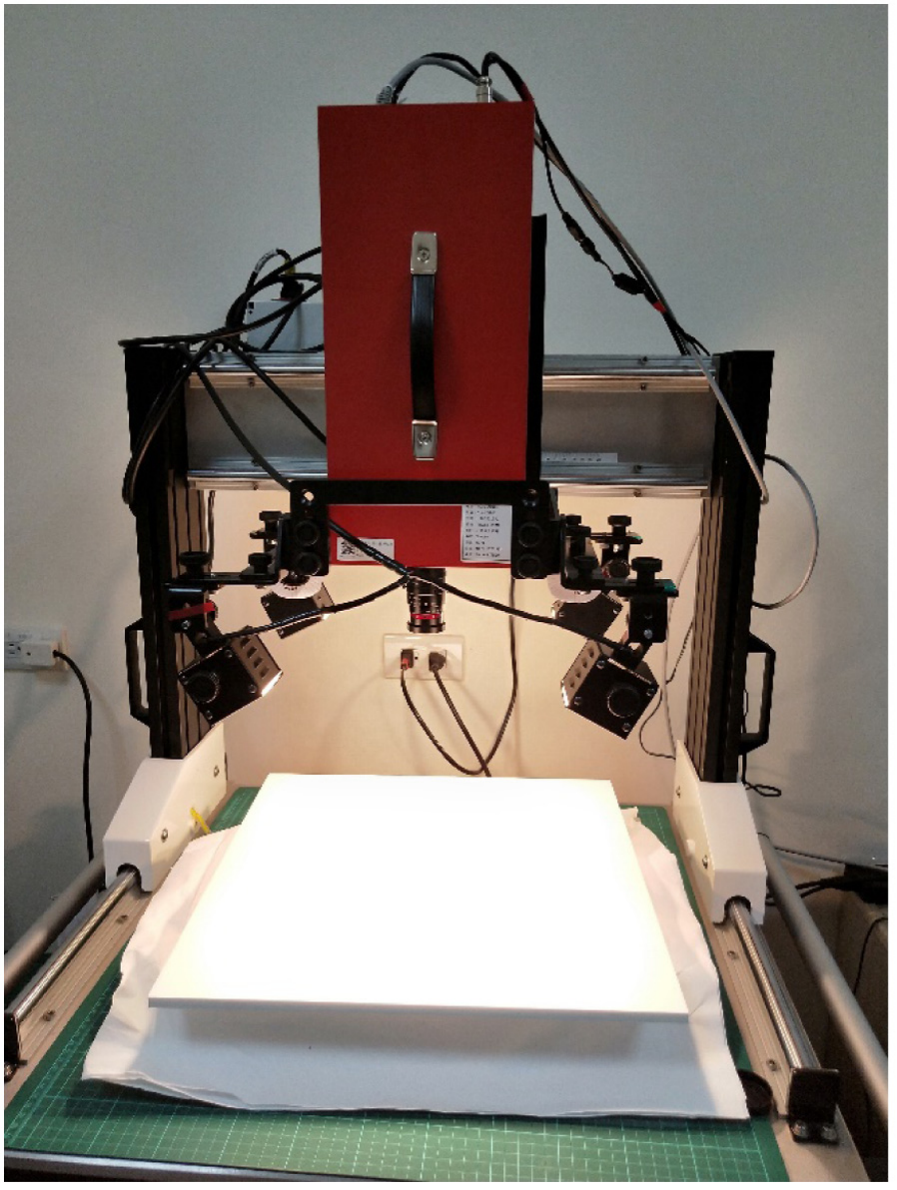
Diagram of short-wave infrared hyperspectral imager and imaging platform used in this study.

The SWIR HSI used in this study employed a near-infrared spectrograph (ImSpector N17E; SPECIM, Oulu, Finland) for spectral separation and an internal scanning device to image the samples. An InGaAs detector (OW1.7-VS-CL-640; Raptor Photonics, Leuven, Northern Ireland) was used to obtain spectral signals of the samples. The spectral range of the sensor covers 900 to 1700 nm, with a spatial resolution of 640×512  pixels and a spectral resolution of 5 nm (with a 30-μm slit). In total, 512 spectral bands were obtained. The imaging system was equipped with four 50-W halogen lamps (51 ALU 41871 WFL 50 W; DecoStar, Osram, Germany). The SWIR-HSI system was mounted on a dedicated platform (Fig. 1).

### 2.2 Phantom Fabrication for Experiment

In this study, agar powder (American Bacteriological Agar 1802; Condalab, Madrid, Spain) and distilled water were used to create collagen phantoms. Depending on the required volume and concentration for the different experiments, the agar powder was mixed with distilled water and heated in a microwave oven until the solution became transparent. The solution was then poured into custom-designed three-dimensionally (3D) printed molds and allowed to solidify.

Details of the designed experimental phantom, 3D printing of the phantom molds, and preparation of the experimental phantoms are provided below.

#### 2.2.1 Experiment 1: ability of SWIR HSI to penetrate collagen phantom samples

**Fig. 2:**
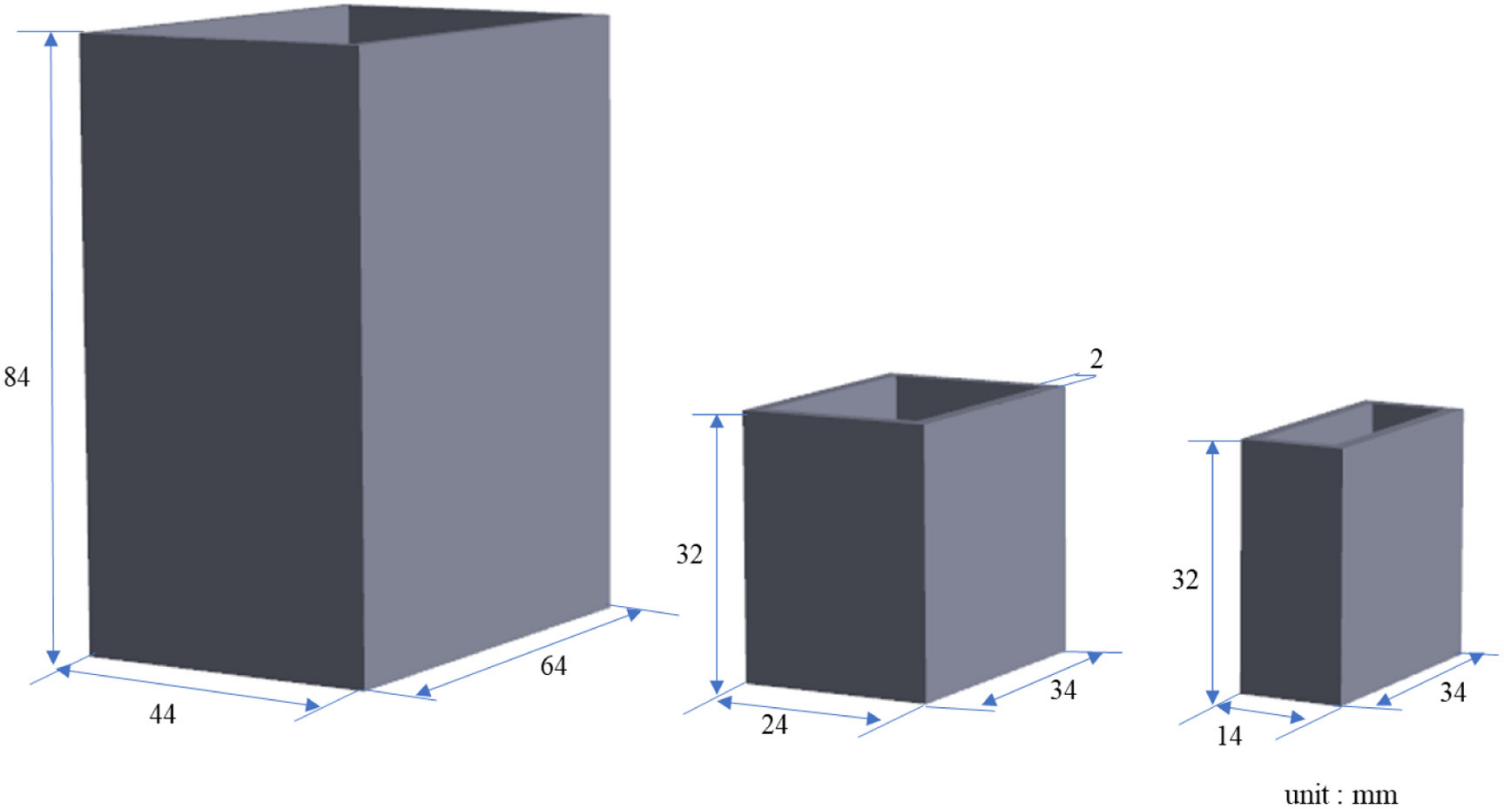
Three-dimensional design diagram of molds for collagen phantoms with different thicknesses.

To understand the ability of SWIR HSI to penetrate the samples, we first designed three molds of different sizes (Fig. 2): 44×64×84, 24×34×32, and $14\times 34\times 32\;{\mathrm{mm}}^{3}$. These molds were 3D-printed at the 3D printing center of our hospital. A 4% collagen solution was prepared, poured into three molds, and left to cool and solidify. Once solidified, the collagen phantoms were removed for SWIR HSI. The actual sizes of the three extracted collagen phantoms were 40×60×80, 20×30×30, and $10\times 30\times 30\;{\mathrm{mm}}^{3}$. These collagen phantoms were used to capture hyperspectral images with six different thicknesses: 10, 20, 30, 40, 60, and 80 mm. Pseudocolor images composed of bands within the NIR-II biological window (1000 to 1350 nm) mentioned in the literature^23^ were used to observe the penetration ability of SWIR HSI through the phantoms. According to a previous study,^23^ this spectral range of the NIR-II window allows better light penetration owing to lower absorption by biological tissues, facilitating signal detection in deeper tissues. Furthermore, there is less interference from the background light in the NIR-II window, such as autofluorescence or other external light sources.

Based on the literature,^23^ we selected the 1091-, 1211-, and 1318-nm bands from the NIR-II biological window to create SWIR pseudocolor images, which were then compared with standard visible color images to observe the penetration ability of SWIR light through collagen phantoms.

#### 2.2.2 Experiment 2: validation of ability of the CEM method to detect subpixel targets at different phantom depths

**Fig. 3:**
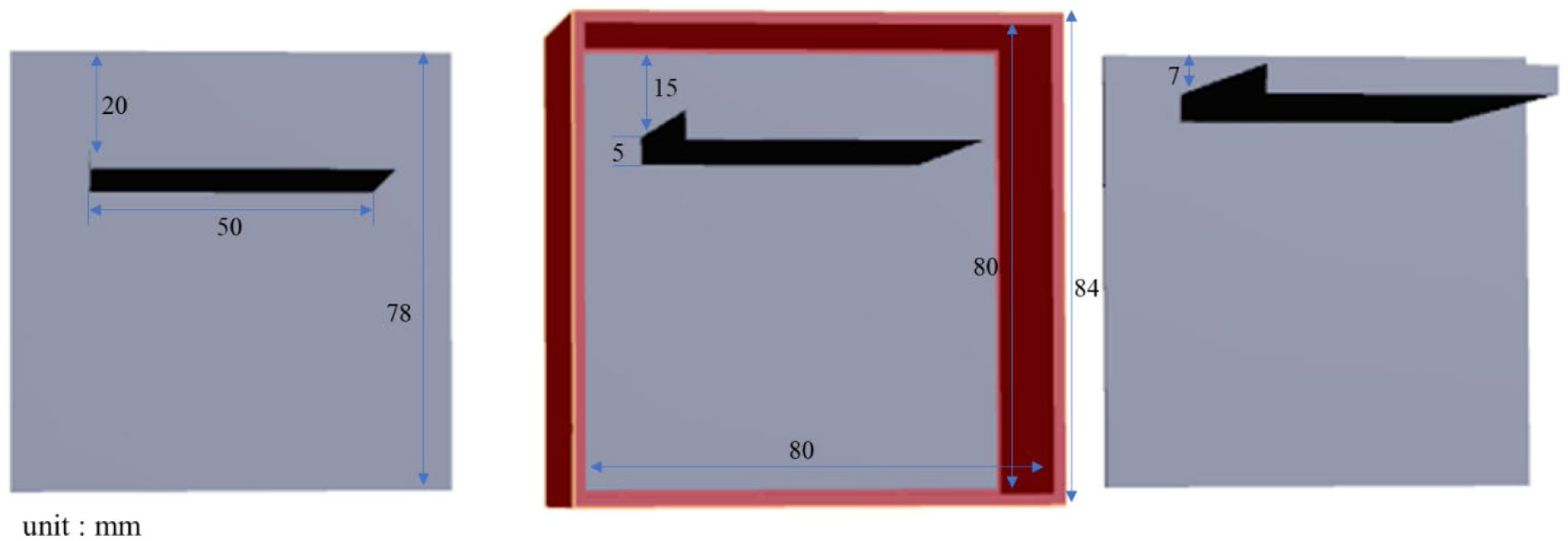
Three-dimensional design diagram of molds for lard–collagen mixed phantoms at different depths.

In this study, 3D printing was used to create molds that allowed the placement of lards (lipids) at three depths (i.e., 7, 15, and 20 mm) below the surface of a collagen phantom. The dimensions of the outer mold were $84\;\mathrm{mm}\times 84\times 62\;{\mathrm{mm}}^{3}$ (Fig. 3). A 4% collagen solution was prepared and poured into three 3D-printed molds. After cooling and solidification, the collagen phantoms were removed from the molds, resulting in final dimensions of $80\;\mathrm{mm}\times 80\times 80\;{\mathrm{mm}}^{3}$.

After removing the collagen phantoms, pure lard was poured into the grooves of the collagen and allowed to cool and solidify. SWIR hyperspectral images were obtained from three lard (lipid)-collagen mixed phantoms on both sides. These produced hyperspectral images of lards at six depths (i.e., 7, 15, 20, 55, 60, and 68 mm). These images allowed us to verify the accuracy of the STD and CEM methods for detecting lard at various depths within the collagen.

We used the known positions of the lard as the ground truth (GT) to verify the reliability and accuracy of the CEM for detecting targets at different phantom depths. The results were compared with a binarized abundance fraction map produced using the CEM. The accuracy of the CEM in detecting the target (lard) was evaluated using the Tanimoto index (TI).^24^ Equation (1) gives TI

$$\mathrm{TI}=\frac{n_{X\cap Y}}{n_{X}{+n}_{Y}{-n}_{X\cap Y}}=\frac{n_{X\cap Y}}{n_{X\cup Y}}.$$ (1)

Let X and Y be two datasets, where X is the binarized result from the CEM method and Y is the GT.

Here, $n_{X}$ represents the number of elements in set X, $n_{Y}$ represents the number of elements in set Y, $n_{X\cap Y}$ represents the union of sets X and Y. A TI value of 0 indicates that X and Y are completely different, whereas a TI value of 1 indicates that the two sets are identical.

#### 2.2.3 Experiment 3: validation of LSU technique for mixed pixels in collagen phantoms at different concentrations and in lard–collagen phantoms with slope distributions

**Fig. 4:**
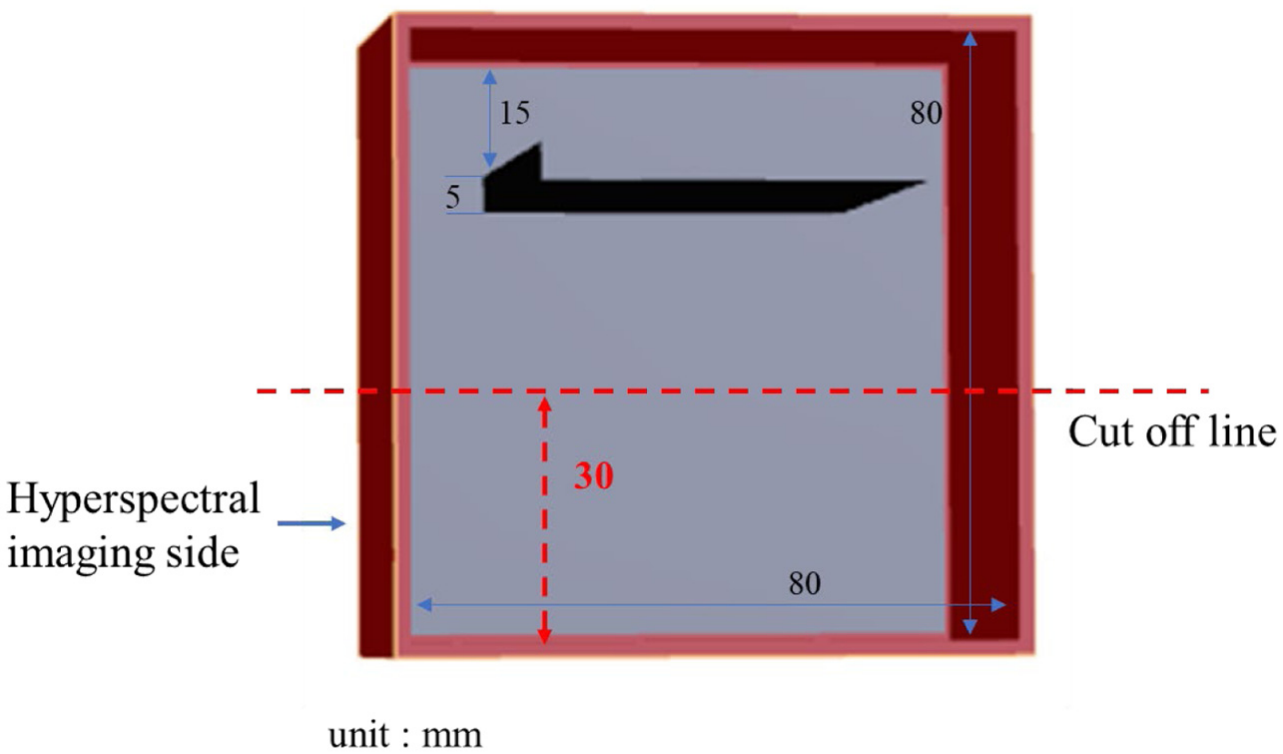
Three-dimensional schematic of the molds used for collagen phantoms with different concentrations.

In this study, four collagen phantoms at different concentrations (2%, 3%, 4%, and 5%) were created using molds from experiment 2. The grooted parts of the collagen phantoms were removed, leaving intact portions for HSI. The volume of each collagen phantom was $80\times 30\times 60\;{\mathrm{mm}}^{3}$ (Fig. 4). Phantoms at four different concentrations were imaged using SWIR HSI. These data were used to verify the accuracy of the LSU-FCLS method in estimating the abundance of collagen at different concentrations.

**Fig. 5:**
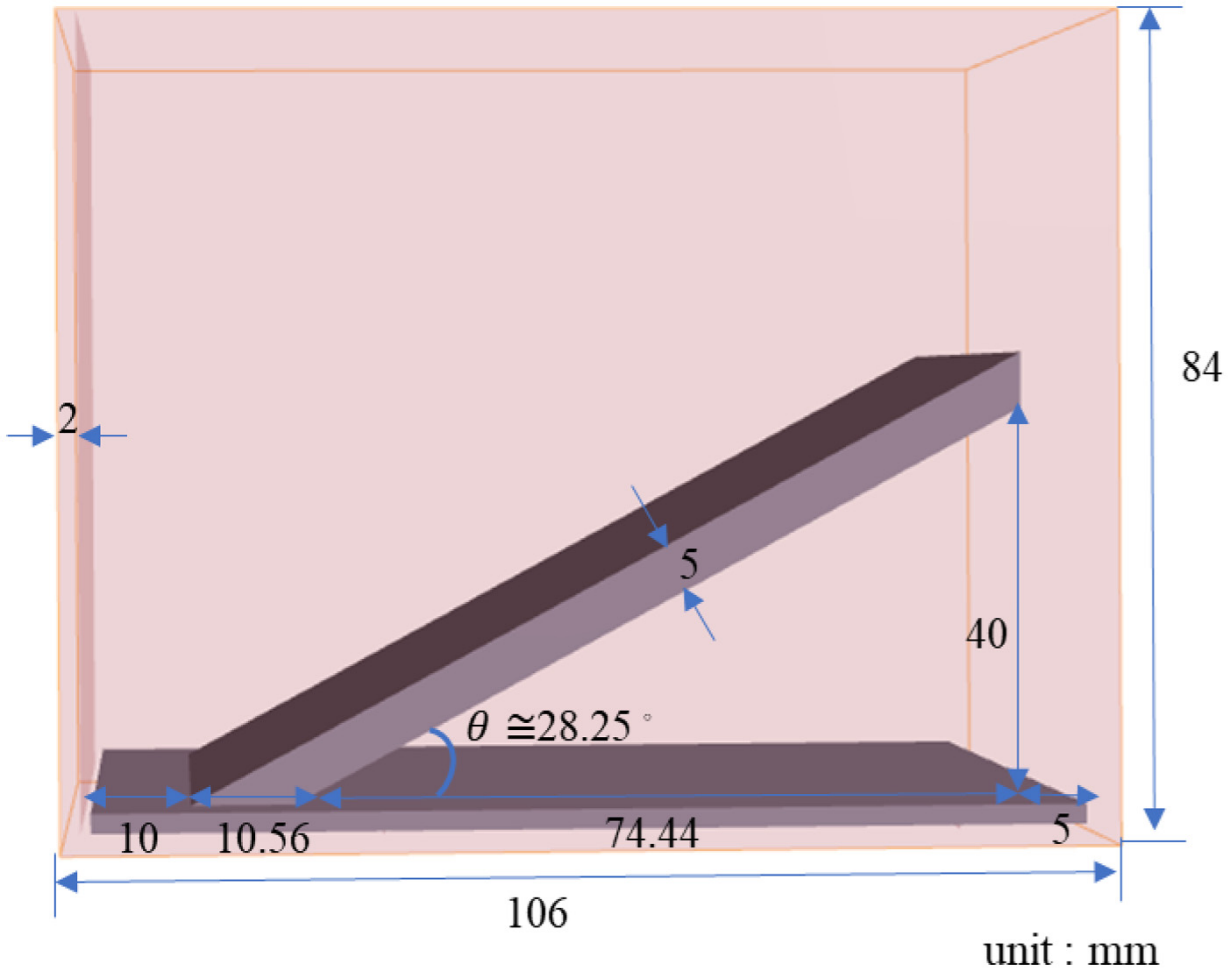
Three-dimensional schematic of the molds used for lard–collagen phantoms with slope distributions.

To further understand the performance of the LSU-FCLS method in determining the sloped lipid (lard) distribution within the same collagen, a phantom was designed, as shown in Fig. 5. The lipids were evenly distributed in the collagen at a slope of ∼28.25  deg, with the greatest vertical height in the collagen reaching 40 mm. This phantom allowed us to investigate whether the distribution results of the endmembers obtained using the FCLS method showed a linear correlation with different depths, thereby verifying the reliability of the FCLS method.

#### 2.2.4 3D printing of phantom molds

The computer model designed using 3D modeling software was saved as a stereolithography file and imported into a vat photopolymerization 3D printer (Form 3; Formlabs, Somerville, Massachusetts, United States) for printing. The 3D printing material used in this study was Formlabs High Temp V2 Resin. The printer was set to a layer height of 0.1 mm, with all other settings left as default. After printing, the object was immersed in 99% isopropyl alcohol for 6 min to remove excess resin from the surface. The object was then air-dried and placed in a temperature-controlled curing machine for post-curing. The post-curing process was set to 80°C for 120 min and repeated twice.

#### 2.2.5 Preparation of experimental phantoms

**Fig. 6:**
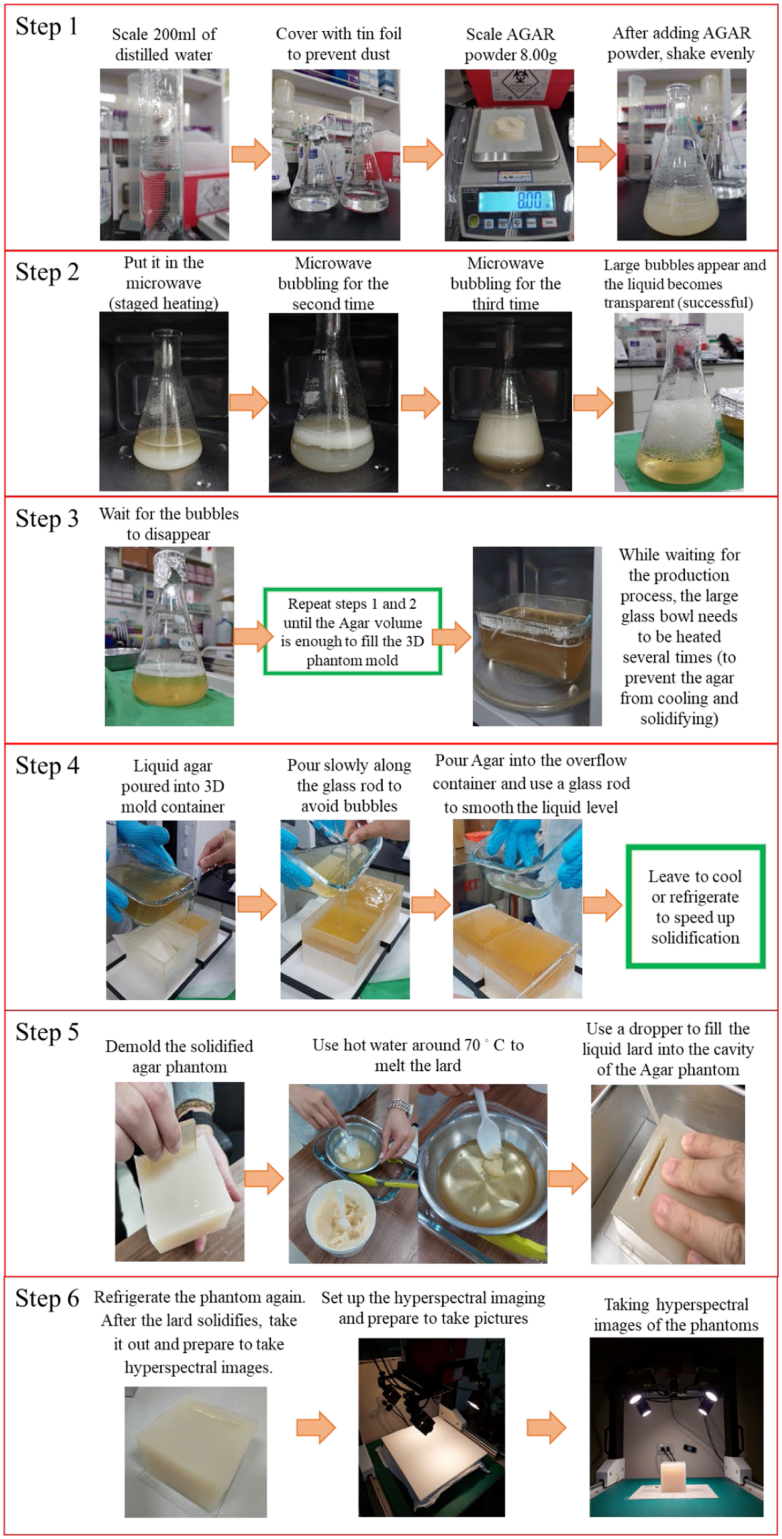
Schematic diagram of the six steps in making an experimental phantom, taking the production of 4% collagen–lard phantom as an example.

After creating the experimental phantom molds using 3D printing (as described in Sec. 2.2.5), this study used agar powder (American bacteriological agar 1802; Condalab, Madrid, Spain), distilled water, and pure lard (purchased from a food supplier, containing 99.78 g of fat per 100 g, with no additives or preservatives) to produce the experimental phantoms. Compared with the other collagen-like materials tested, agar powder is easier to demold from 3D molds after solidification and is less prone to deformation. The process of phantom fabrication and hyperspectral imaging included six main steps. Using the preparation of a 4% collagen–lipid phantom as an example, the steps are detailed below, and Fig. 6 illustrates the experimental process.

Step 1: For the agar mixing step, 200 mL of distilled water and 8.00 g of agar powder were measured and poured into a conical flask, mixing evenly by shaking.
Step 2: For the heating step, a conical flask was placed in a microwave and heated at intervals of 45 s at medium power, repeating four to five times until the agar solution became transparent. The microwave used in this study was a Panasonic NN-SM33NW with five power settings: low, defrost, medium, medium-high, and high.
Step 3: Steps 1 and 2 were repeated. It is not recommended to prepare more than 250 mL of agar solution at a time, as it may overflow during heating and prolonged heating may affect the quality of the solution. Therefore, steps 1 and 2 were repeated multiple times, and the prepared agar solution was poured into a large glass bowl after each batch until the required volume was reached. During this process, the agar solution in the glass bowl was reheated periodically in a microwave to prevent solidification.
Step 4: For the filling and cooling step, the liquid agar was slowly poured into the 3D molds along a glass rod to avoid creating bubbles during pouring. After filling, the molds were left to cool naturally or refrigerate to speed up solidification.
Step 5: For demolding and lard filling, once the agar had solidified (∼30  min in the refrigerator), the agar phantom was removed from the mold. Next, the solid lard was melted using a water bath at ∼70°C. Liquid lard was drawn with a dropper and used to fill the agar phantom. After filling, the phantom was refrigerated for ∼5  min to accelerate the lard solidification.
Step 6: For the hyperspectral imaging step, the phantom was removed from the refrigerator once the lard had solidified. Last, the hyperspectral imaging system was prepared, and hyperspectral images of the experimental phantom were captured.

### 2.3 Hyperspectral Image Processing

#### 2.3.1 CEM STD method

**Fig. 7:**
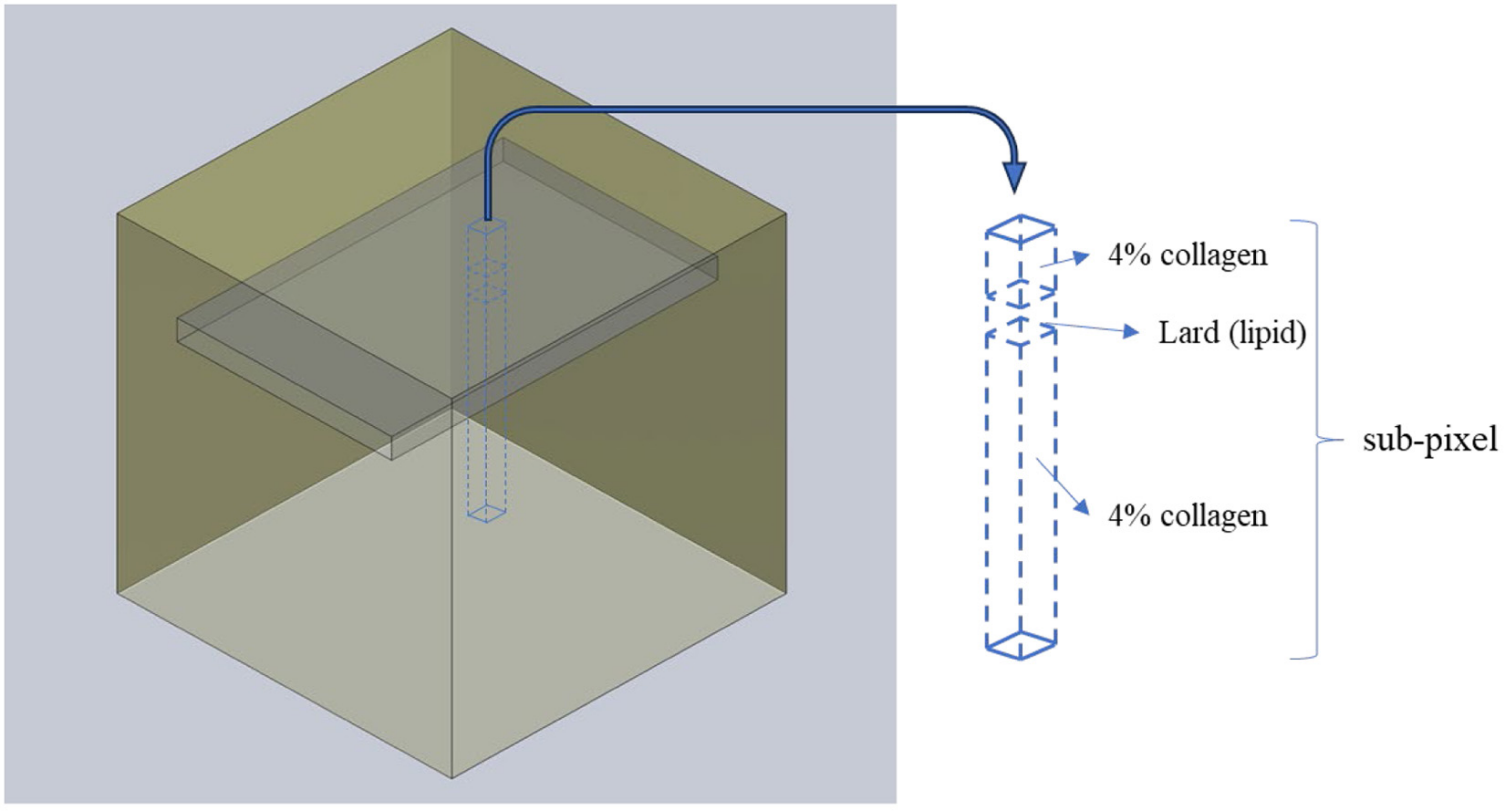
Concept diagram of subpixels in the hyperspectral image of the lard (lipid)–collagen mixed phantom.

In hyperspectral image processing, a subpixel refers to a target or feature that is smaller than a single pixel. Because each pixel in a hyperspectral image contains rich spectral information, even if the target is too small to occupy a full pixel, it can still be identified based on its unique spectral features. In this study, the lard (lipid)–collagen mixed phantom we created had lard embedded within the collagen (Fig. 7). Therefore, the lard (target) is a subpixel in hyperspectral image processing.

In this study, we used CEM, the most commonly applied method for STD in previous studies,^25^ to analyze and detect the target of interest (lard). The basic concept involves the design of a finite impulse response (FIR) filter based on the spectrum of the target material. When the input spectral vector is multiplied by this filter vector, the other spectral vectors are suppressed, and the target of interest is highlighted. If the spectral vector is similar to the target, the output value is close to 1; otherwise, it approaches 0. In this study, we used the CEM method to detect lard embedded at different depths in collagen phantoms to verify the ability of the CEM method to detect targets (lard) at various depths.

Let L be the dimension of the spectral bands and $r=\{r_{1},r_{2},\ldots ,r_{N}\}$ represent the set of all pixel sequences in the hyperspectral image, where the i’th pixel vector is $r_{i}=(r_{i1},r_{i2},\ldots ,r_{iL}{)}^{T}$ and N is the total number of pixels in the image. Here, we assume that the spectral signal of the target of interest (lard) is $d=(d_{1},d_{2},d_{3},\ldots ,d_{L}{)}^{T}$, and we designed an L-dimensional linear FIR filter for the target, which we denote as $w=(w_{1},w_{2},w_{3},\ldots ,w_{L}{)}^{T}$. The spectral vector of the target has a minimized output after passing through the FIR filter and is subject to the condition of $d^{T}w=w^{T}d=1$.

We assume that $y_{i}$ is the output result of the i’th pixel spectrum ($r_{i}$) in the hyperspectral image after passing through the FIR filter, as shown in Eq. (2)

$$y_{i}=\overset{L}{\underset{l}{\sum }}w_{l}r_{il}=w^{T}r_{i}.$$ (2)

Thus, we can calculate the average energy result for all pixels $\left\{r_{1},r_{2},\cdots ,r_{N}\right\}$ as shown in Eq. (3)

$$\frac{1}{N}[\overset{N}{\underset{i=1}{\sum }}y_{i}^{2}]=w^{T}R_{L\times L}w,$$ (3)

where $R_{L\times L}=\frac{1}{N}[\overset{N}{\underset{i=1}{\sum }}r_{i}r_{i}^{T}]$ is the autocorrelation matrix of all pixel points in the hyperspectral image. Therefore, the CEM used herein aims to solve the linear constrained minimization problem, as shown in Eq. (4)

$$\min_{W}\;\{w^{T}R_{L\times L}w\}\;\text{subject to }d^{T}w=1.$$ (4)

According to a previously described derivation,^26^ the optimal solution to Eq. (4) is given by Eq. (5)

$$w^{*}=\frac{R_{L\times L}^{-1}d}{d^{T}R_{L\times L}^{-1}d}.$$ (5)

Finally, we used the optimized weight matrix from Eq. (5) to detect lard targets. The resulting abundance matrix was denoted as A, as shown in Eq. (6). The result of Eq. (6) was an abundance map of the target (lard)

$$A=(w^{*}{)}^{T}r=\frac{d^{T}R_{L\times L}^{-1}r}{d^{T}R_{L\times L}^{-1}d}.$$ (6)

#### 2.3.2 LSU-FCLS method

**Fig. 8:**
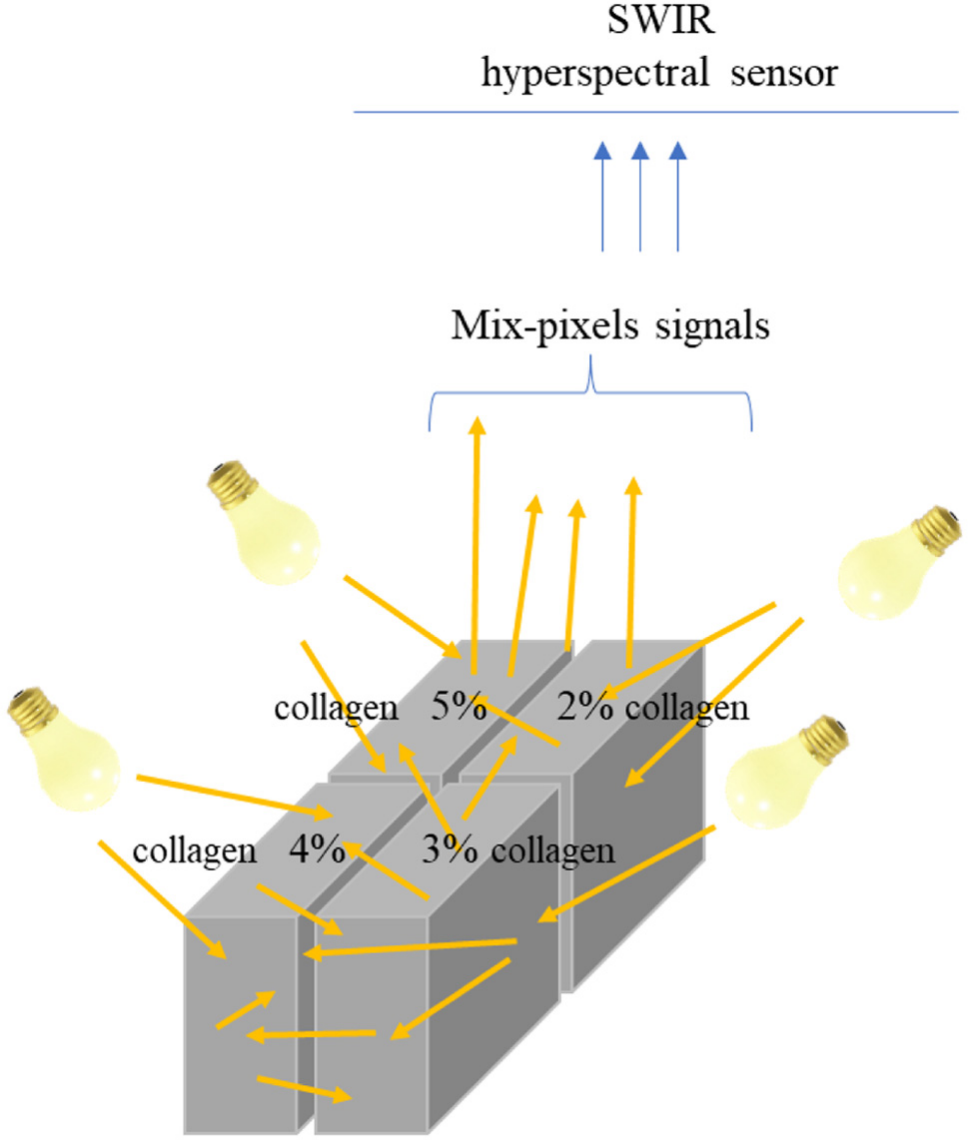
Concept diagram of the hyperspectral imaging setup and mixed pixels for four collagen phantoms with different concentrations. This figure assumes that short-wave infrared light can penetrate collagen and is used to represent the concept of mixed pixels to demonstrate the application of the LSU-FCLS method in spectral unmixing, which is different from sub-pixel detection.

The purpose of the setup was to demonstrate the concept of mixed pixels and validate the performance of the LSU-FCLS method for spectral unmixing (Fig. 8). SWIR light can significantly penetrate the collagen phantoms, leading to the assumption that the hyperspectral signal received by the sensor represents a linear combination of spectral signals from the four collagen phantoms at different concentrations (2%, 3%, 4%, and 5%). The experimental setup focused on simulating mixed-pixel conditions rather than subpixel-level detection, aiming to evaluate the capability of LSU-FCLS in unmixed mixed-pixel spectral data and to accurately estimate the abundance of different collagen concentrations. This study acknowledges that this design does not directly address the potential spectral crosstalk effects caused by adjacent phantoms. Future work will include experiments comparing hyperspectral signals from single and multiple phantoms under identical imaging conditions to further investigate neighboring phantom effects.

The FCLS algorithm,^27^ which is widely used in LSU for HSI, was applied to analyze the SWIR hyperspectral images of four collagen phantoms with different concentrations. This method can accurately estimate the abundant content for each concentration, thereby verifying the feasibility of clinical hyperspectral image analysis applications. The following is an introduction to the FCLS algorithm:

First, we assumed that the pixel signal in the hyperspectral image was a linear mixture of signals from the four different concentrations of collagen phantoms. In this case, the collagen phantoms are composed of p endmembers. Therefore, we can represent the pixel spectral signal in the image as a combination of p endmembers $\left(m_{1},m_{2},\cdots ,m_{p}\right)$, and it can be written as $M=[m_{1},m_{2},\cdots m_{p}]$. In addition, let the corresponding weights for the linear mixture of these endmembers be $\alpha =[\alpha_{1},\alpha_{2},\cdots \alpha_{p}{]}^{T}$. The linear mixture can be expressed mathematically as shown in Eq. (7)

r=Mα+n. (7)

Here, n represents the noise vector, whereas $\alpha_{j}$ is the abundance fraction of the j’th endmember ($m_{j}$) in the pixel vector r. To solve the linear mixture model in Eq. (7), one of the most commonly used methods is the least squares error approach, which is solved by $\min_{\alpha }\{(r-M\alpha {)}^{T}(r-M\alpha )\}$. The optimal solution can be obtained using Lagrange multipliers, as expressed in Eq. (8)

α^LS(r)=(MTM)−1MTr. (8)

Here, α^LS(r)=(α^1LS(r),α^2LS(r),…α^pLS(r)), where α^jLS(r) represents the abundance fraction of the j’th endmember $m_{j}$ estimated from the sample vector r.

However, the α^LS(r) value obtained from Eq. (8) does not satisfy the abundance constraints. Here, we will add two constraints: the abundance sum must equal 1 ($\sum_{j=1}^{p}\alpha_{j}=1$)), and the abundance must be greater than or equal to 0 (α≥0). This method is referred to as the FCLS. In this study, the spectral unmixing model for collagen at different concentrations aimed to determine the optimal solution to Eq. (9), thereby estimating the abundance of collagen in phantoms with varying concentrations

$$\min_{\alpha }\{(r-M\alpha {)}^{T}(r-M\alpha )\}\;\text{subject to }\overset{p}{\underset{j=1}{\sum }}\alpha_{j}=1\text{ and }\alpha \geq 0.$$ (9)

The optimal solution of Eq. (9) can first be obtained using the solution α^LS(r) from Eq. (8) as the initial estimate. The optimal solution can then be determined using the Lagrange multipliers, as shown in Eq. (10)

α^FCLS(r)=PM,1⊥α^LS(r)+(MTM)−11[1T(MTM)1]−1. (10)

Here, $P_{M,1}^{⊥}=I_{L\times L}-(M^{T}M{)}^{-1}1[1^{T}(M^{T}M)1{]}^{-1}1^{T}$, I is an identity matrix. A more detailed derivation of this equation can be found elsewhere.^28^

## 3 Results

### 3.1 Ability of SWIR HSI Technology to Penetrate Collagen Phantom Samples

In this study, we used spectral bands from the NIR-II biological window mentioned elsewhere^23^ to create pseudocolor images and observed the ability of SWIR HSI to penetrate collagen phantoms of different thicknesses. Because the grid lines in the background are no longer visible when the collagen phantom thickness exceeds 60 mm, we present only the results up to 60 mm.

**Fig. 9:**
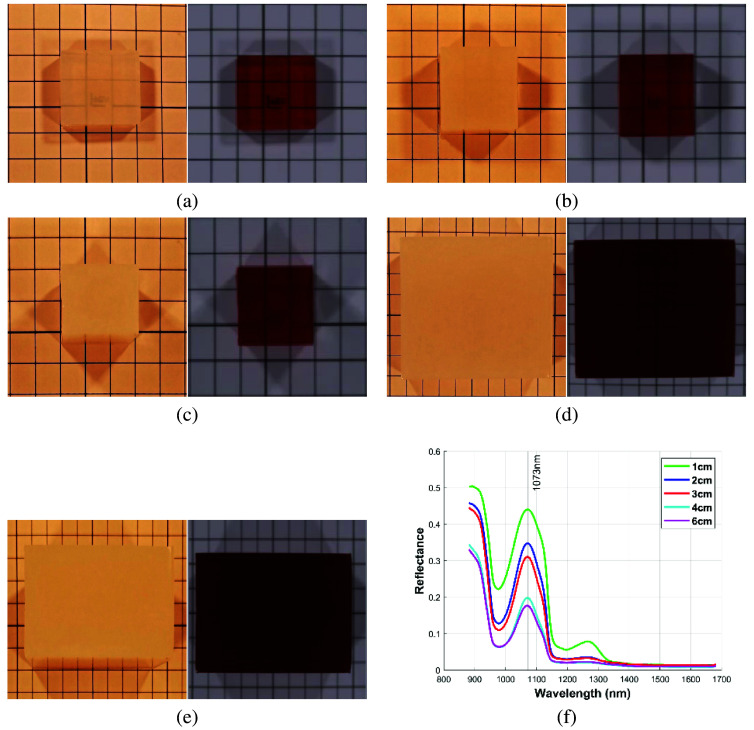
Color images and short-wave infrared pseudocolor images of collagen phantoms with different thicknesses: (a) 10 mm, (b) 20 mm, (c) 30 mm, (d) 40 mm, and (e) 60 mm; (f) average reflectance spectral profile of collagen phantoms with different thicknesses.

Figures 9(a)–9(e) show the visible-color images and SWIR pseudocolor images of the collagen phantoms with thicknesses of 10, 20, 30, 40, and 60 mm, respectively. Figure 9(f) shows the average spectral profiles of the five collagen phantoms with different thicknesses. Figures 9(a)–9(e) show that the visible color images are unable to show the background grid when the thickness of the collagen phantom exceeds 10 mm. By contrast, the SWIR pseudo-color images displayed a background grid for collagen thicknesses of 10 to 30 mm. At 40 mm, the grid is barely visible and blurry, whereas at 60 mm, the background grid is no longer visible.

The past literature^8^ pointed out that at a wavelength of ∼1050  nm, there is a local minimum for water and a local maximum for collagen absorption coefficients, making this wavelength range suitable as a spectral window for collagen detection. As shown in the spectral profiles in Fig. 9(f), the thickness of the collagen phantoms increased, and the measured spectral reflectance at 1073 nm decreased accordingly.

### 3.2 STD: Ability of CEM Method to Detect Targets in Phantoms with Different Depths

**Fig. 10:**
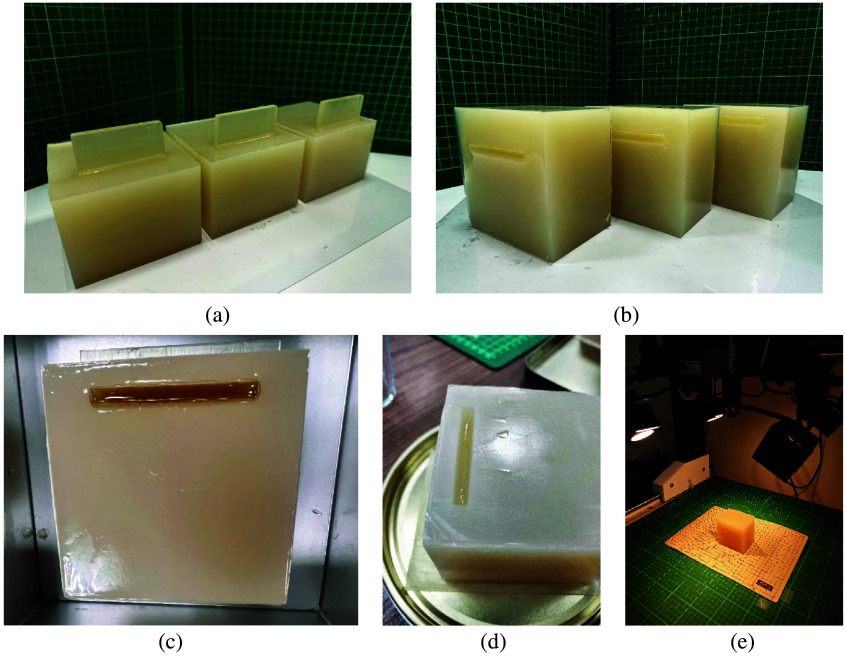
(a) Creating grooves at different depth positions in the collagen phantom using a three-dimensionally printed mold (before demolding), (b) collagen phantom after demolding, (c) injection of pure lard into the grooves of the collagen phantom, (d) lipid–collagen mixed phantom after the lard has cooled, and (e) capturing short-wave infrared hyperspectral images of the lipid–collagen mixed phantom.

Based on the results of the experiment described in Sec. 3.1, SWIR HSI can penetrate collagen phantoms up to a thickness of at least 40 mm. However, visual inspection of the SWIR pseudo-color images at 60 mm revealed that penetration was impossible. Therefore, this study used 3D printing to create molds that allowed HSI to occur at six different depths: 7, 15, 20, 55, 60, and 68 mm. A 4% collagen phantom was first made and removed from the mold [Figs. 10(a) and 10(b)], and the grooves were then filled with pure lard (lipid) to create a lipid–collagen mixed phantom [Fig. 10(c)]. After the lard (lipid) solidified [Fig. 10(d)], SWIR hyperspectral images were captured [Fig. 10(e)].

**Fig. 11:**
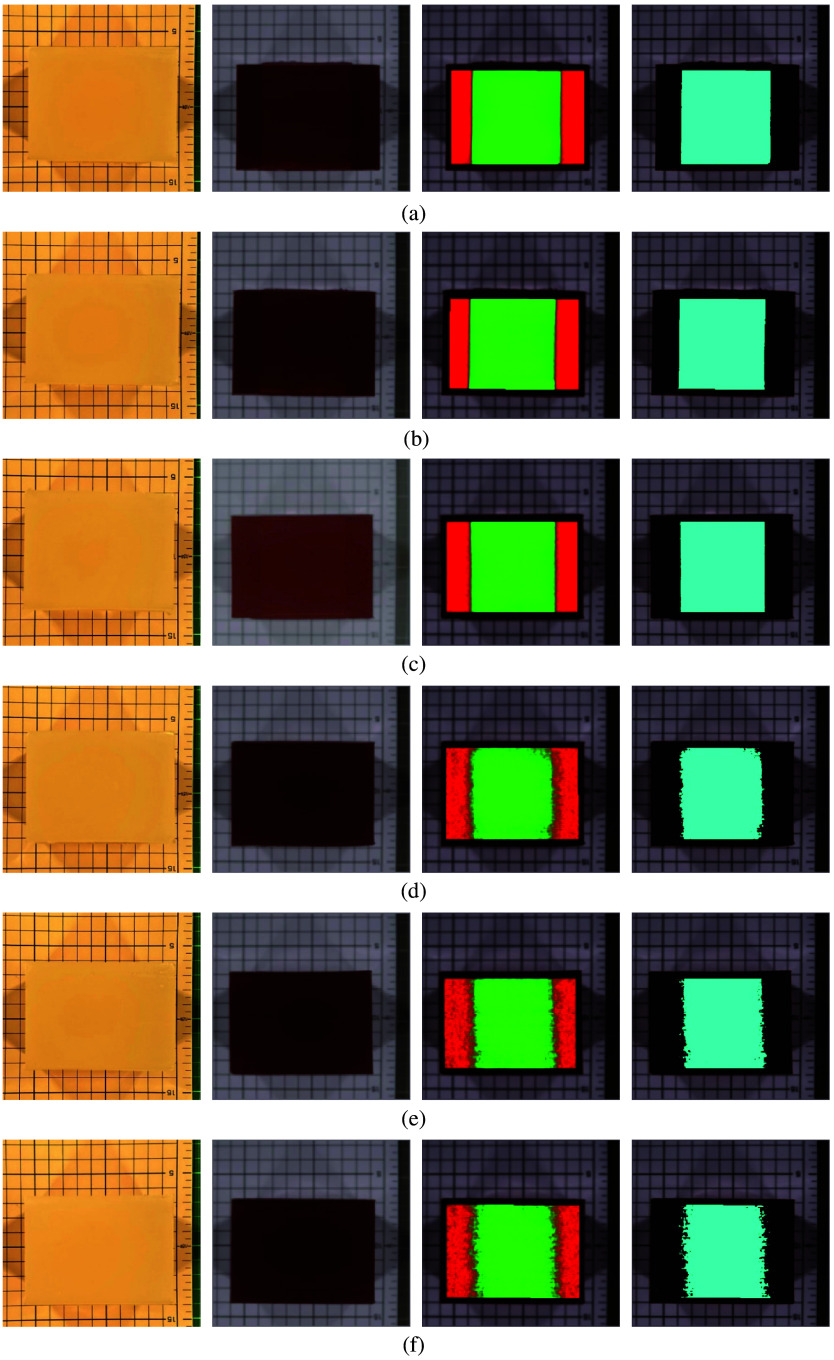
Each row, from left to right, represents the visible color image, short-wave infrared pseudocolor image, constrained energy minimization abundance map, and binarized abundance map using Otsu’s method for the lipid–collagen mixed phantom with lards embedded at different depths: (a) 7 mm, (b) 15 mm, (c) 20 mm, (d) 55 mm, (e) 60 mm, and (f) 68 mm.

Figure 11(a) shows, from left to right, the visible color image of the lipid–collagen mixed phantom with the lard embedded at a depth of 7 mm, the SWIR pseudocolor image, the abundance map of the CEM method (green represents the detected lard, and red represents the background), and the binarized result of the abundance map using Otsu’s method^29^ (represented in light blue). Figures 11(b)–11(f) show the experimental results for the lipid–collagen mixed phantoms at depths of 15, 20, 55, 60, and 68 mm, respectively.

Figure 11 shows that it was impossible to identify whether pure lard was embedded under collagen in the visible color images of the lipid–collagen mixed phantoms at different depths, even at 7 mm. However, in the SWIR pseudocolor images, it was barely possible to see other substances inside the collagen phantom at depths of 7 and 15 mm. However, the abundance maps clearly show that the CEM method can easily detect embedded lards at depths of 7, 15, and 20 mm. At depths of 55, 60, and 68 mm, as the lard embedding depth increased, the accuracy of the CEM method for detecting lard decreased, particularly at the boundary between the lard and the collagen phantom.

**Fig. 12:**
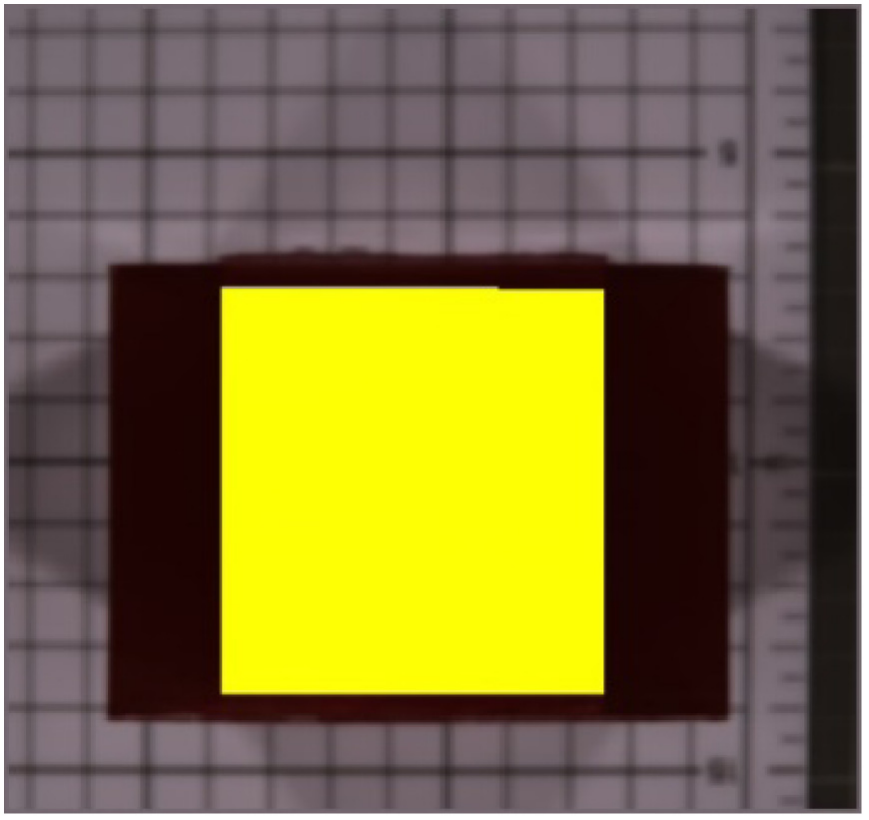
Ground-truth image of the embedded lard positions.

This study further calculated the Tanimoto index (TI) by comparing the binarized results of the CEM method with the GT of the embedded lard positions, as shown in Fig. 12. Table 1 shows that the TI values for the CEM method remain above 0.98 at depths of 7, 15, 20, and even 68 mm, and the TI value remains above 0.90. These results demonstrate that the CEM method can effectively detect targets at different depths in SWIR hyperspectral images, providing better detection accuracy than the visual results from visible-color images and SWIR pseudocolor images.

**Table 1:** Results of the TI calculation using the binarized results of constrained energy minimization and ground truth.

Depth of embedded lard (mm)	7	15	20	55	60	68
TI	0.988	0.985	0.988	0.932	0.924	0.907

### 3.3 Validation of LSU Technique for Mixed Pixel in Collagen Phantoms with Different Concentrations and in Lard–Collagen Phantoms with Slope Distributions

This experiment aimed to validate the application of the FCLS method in spectral unmixing rather than directly simulating subpixel detection conditions. In this study, four collagen phantoms with different concentrations (i.e., 2%, 3%, 4%, and 5%) were used to verify the accuracy of the FCLS method in estimating the abundance of collagen at different concentrations. From the results in Sec. 3.1, we know that the SWIR pseudocolor images cannot penetrate the bottom of an object when the collagen phantom thickness exceeds 60 mm. Therefore, in this experiment, the heights of the four phantoms with different concentrations were set to 80 mm for SWIR HSI. The main purpose was to ensure that no concentration differences were observed, which allowed us to verify the performance of the proposed FCLS method.

The FCLS method performs unmixing analysis on mixed pixels and estimates the abundance of reference spectral endmembers in the mixed pixels. Because different concentrations of collagen were prepared using only collagen powder and distilled water in varying ratios, this study assumed that collagen at extreme concentrations (2% and 5%) would serve as the first and second endmembers for the spectral unmixing analysis. The FCLS method was then used for the unmixing analysis. The more similar the spectral characteristics of the mixed pixels are to the endmembers, the higher the estimated abundance. To verify the reliability and accuracy of the FCLS method, known concentration ratios were used as the GT to calculate the correlation coefficient between the FCLS estimation results and the GT.

**Fig. 13:**
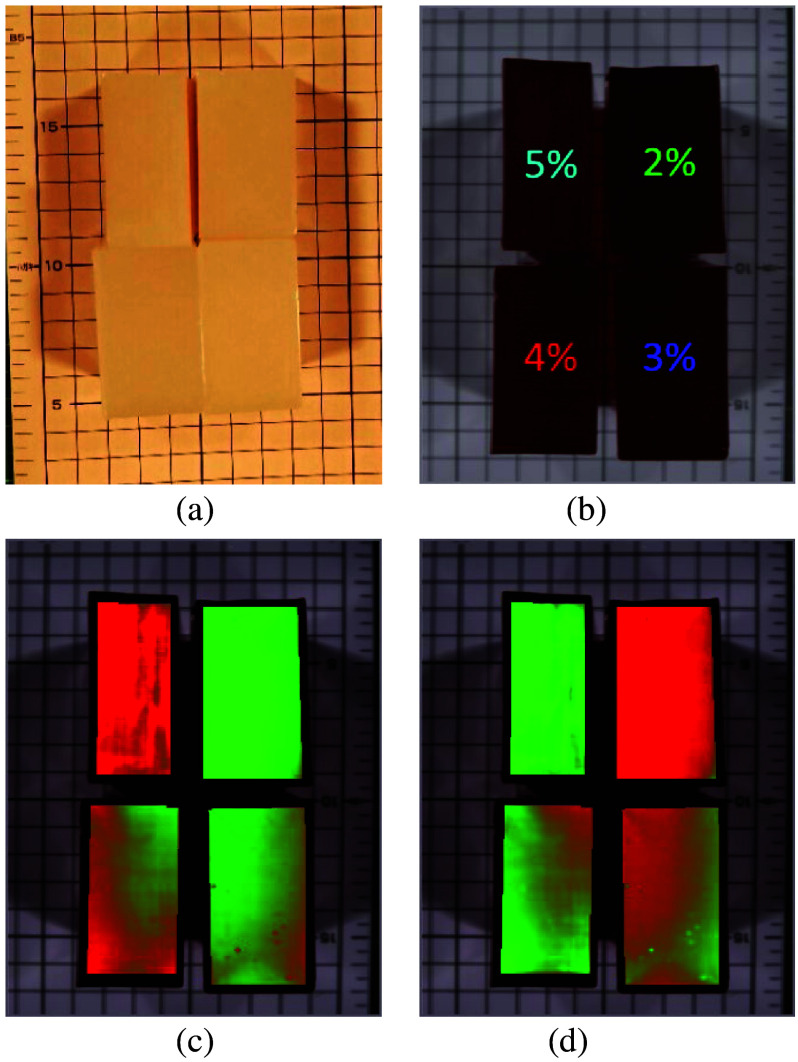
Collagen phantoms with different concentrations: (a) visible color image, (b) short-wave infrared pseudocolor image, (c) fully constrained least squares abundance map using the first endmember (2% concentration), and (d) fully constrained least squares abundance map using the second endmember (5% concentration).

Figures 13(a) and 13(b) show that neither the visible-color image nor the SWIR pseudocolor image could distinguish the concentration differences in the collagen phantoms. Figures 13(c) and 13(d) show abundance maps for the first and second endmembers obtained using the FCLS method. The abundance maps clearly differentiated among the different concentrations of collagen phantoms.

**Fig. 14:**
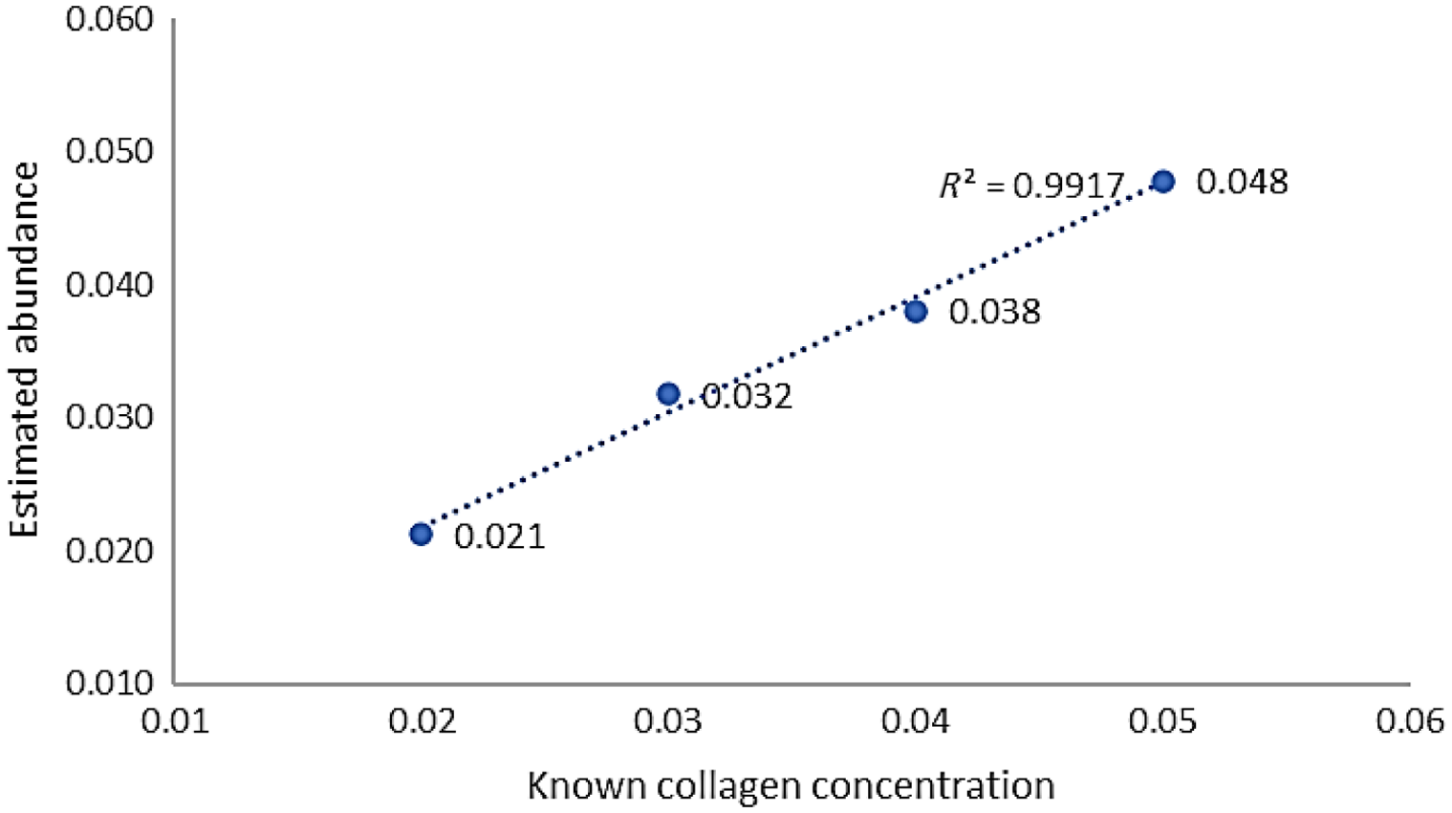
Correlation coefficient results between fully constrained least squares unmixing abundance estimates and ground truth for collagen phantoms with different concentrations.

Table 2 presents the results of the estimation of collagen abundance for the phantoms using the average spectral signals of the 2% and 5% concentrations of collagen phantoms as the first and second endmembers, respectively. A linear correlation analysis of the estimated results and known collagen concentrations showed an $R^{2}$ value of 0.9917 (Fig. 14), confirming that the FCLS method could effectively perform unmixing and estimate the abundance of collagen in phantoms with different concentrations.

**Table 2:** Abundance analysis of collagen phantoms at different concentrations using the fully constrained least squares unmixing method.

Phantoms	2% collagen phantom	3% collagen phantom	4% collagen phantom	5% collagen phantom
Endmember 1 (2% collagen, α^1)	0.957	0.611	0.401	0.078
Endmember 2 (5% collagen, α^2)	0.042	0.391	0.601	0.926

**Fig. 15:**
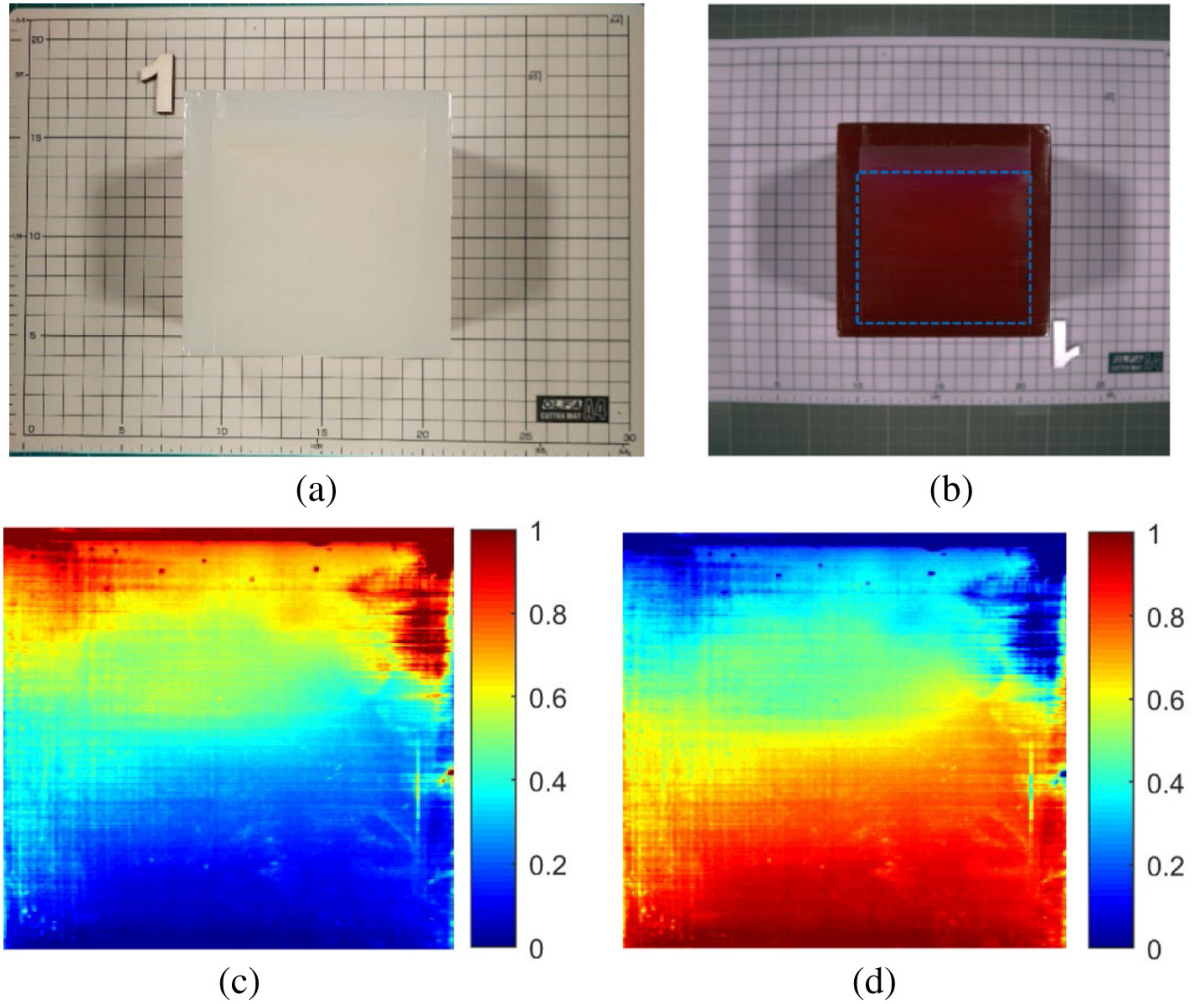
Lard–collagen phantoms with slope distributions: (a) visible color image, (b) short-wave infrared pseudocolor image, (c) distribution fraction of endmember 1 (lard, α^l) using the FCLS method, and (d) distribution fraction of endmember 2 (collagen, α^c) using the FCLS method.

In addition, a collagen phantom with a sloped lard distribution was used to estimate the lipid distribution within the collagen using the FCLS method [Fig. 15(a)]. As we focused only on the distribution of lipids (lard) within collagen, the analysis area was limited to the blue dashed box shown in Fig. 15(b). In this region, the lard was evenly distributed on a 28.25-deg slope within the collagen, and other optical interference was not considered.

For the analysis, the shallowest pixel containing lard (close to 0 cm depth) was used as the first endmember (lard), and the deepest pixel containing lard (4 cm depth) was used as the second endmember (collagen). The FCLS method was used to decompose the data. To verify the reliability of the FCLS in estimating lipid distribution at different depths, the distribution content of the first and second endmembers was calculated for all pixels in the image and their correlations at depths of 1, 2, 3, and 4 cm were analyzed. This study aimed to verify whether the distribution of the first and second endmembers exhibited a linear relationship with depth.

**Fig. 16:**
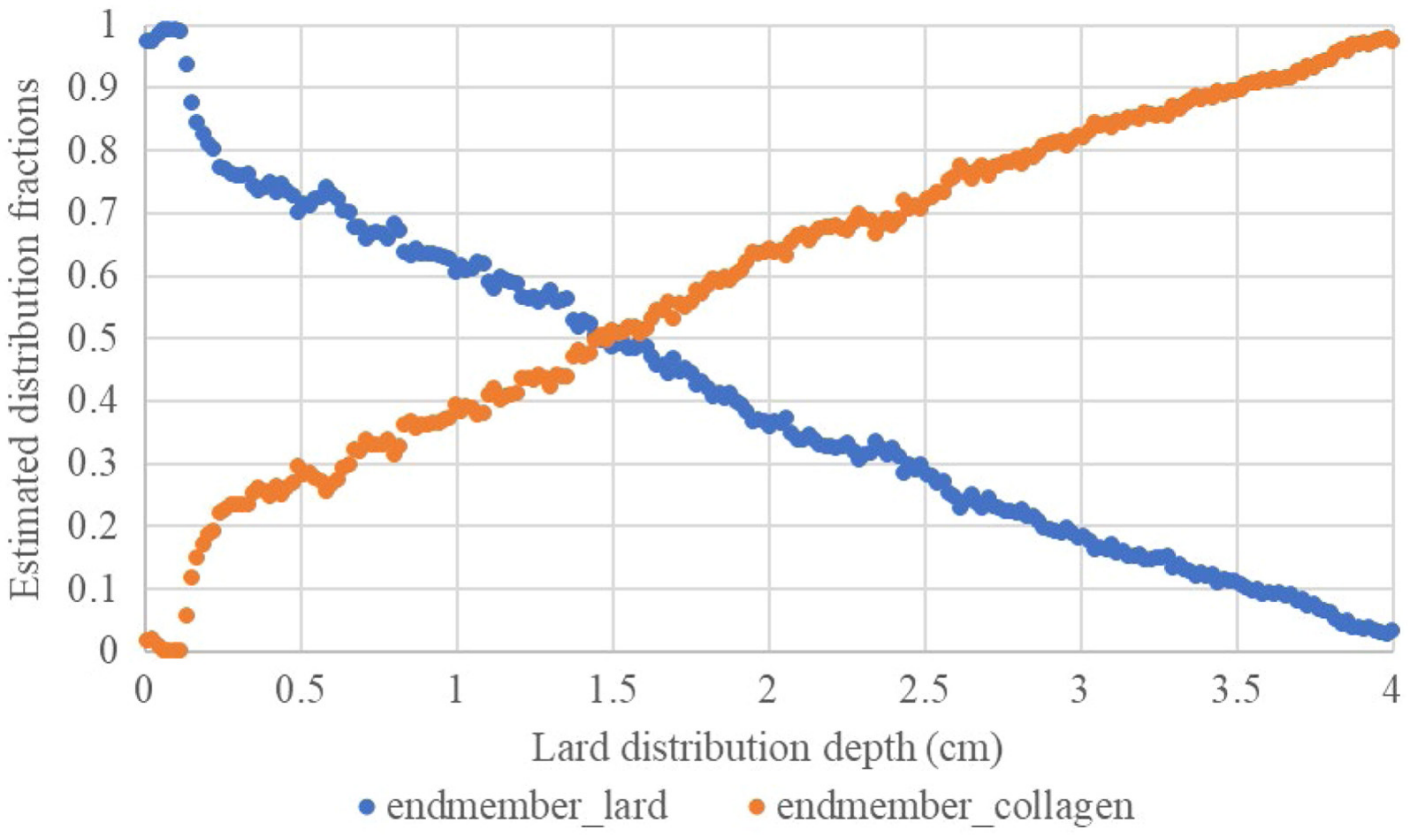
Estimated distribution fractions after averaging the pixels in each column of the two endmembers.

As shown in Figs. 15(c) and 15(d), which present the distribution results of the first and second endmembers obtained using the FCLS method, the distribution of the first endmember decreased from top to bottom, whereas that of the second endmember increased. Figure 16 shows the estimated distribution fractions after averaging the pixels in each column for the two endmembers. The distribution values of these endmembers were analyzed using Spearman’s correlation at depths of 1, 2, 3, and 4 cm. The results indicate that the distribution content changes of the two endmembers extracted by FCLS had a highly linear correlation with depth changes, both exceeding 0.9812 (Table 3). These findings indicate that the FCLS method provides reliable estimates of endmember distribution.

**Table 3:** Distribution fractions of two endmembers at different depths and Spearman’s correlation between each endmember’s distribution fraction and depth.

Depth (cm)	Distribution fraction of endmember 1 (lard, α^l)	Distribution fraction of endmember 2 (collagen, α^c)	Spearman’s correlations (endmember 1)	Spearman’s correlations (endmember 2)
1	0.6061	0.3939	−0.9812**	0.9812**
2	0.3630	0.6370	−0.9963**	0.9963**
3	0.1761	0.8239	−0.9983**	0.9983**
4	0.0257	0.9743	−0.9992**	0.9992**

** p<0.01.

## 4 Discussion

The main results of this study show that through the creation of different simulated biological tissue phantoms and the analysis of SWIR hyperspectral images, the SWIR HIS has a strong penetration ability. This was observed in collagen phantoms at various depths and demonstrated precise STD. We found that the STD technique could effectively identify and detect the target of interest even at a collagen phantom thickness of 60 mm. This has not been previously performed in clinical research using SWIR HSI. Our results also showed that wavelengths in the SWIR range (900 to 1700 nm) have a strong ability to penetrate collagen phantoms while maintaining stable spectral characteristics, enabling reliable imaging at greater depths.

We also used 3D printing to create molds that allowed the embedding of target objects at different depths. This enabled us to produce lipid–collagen mixed phantoms with lards embedded at various depths for SWIR HIS analysis. We verified the performance of STD using the CEM method for lipid detection at different depths. The experimental results showed that the CEM method could detect lipids with high accuracy at depths between 7 and 20 mm, effectively identifying both lipid location and abundance. However, when the embedding depth exceeded 20 mm, the detection accuracy began to decrease, indicating that this method may be more affected by light scattering and absorption when examining deeper tissue components. Therefore, future research may be needed to further explore this method to improve its application in deep-tissue detection.

We also performed LSU analysis of collagen phantoms at different concentrations. The results showed that the FCLS method accurately estimated the abundance within the 2% to 5% concentration range, achieving a correlation coefficient of 0.9917 with the GT. In addition, a collagen phantom mixed with sloped distributions of lard was used to estimate the lipid (lard) distribution within collagen using the FCLS method. The results showed that the two endmember components extracted by FCLS had highly linear correlations with depth changes, both exceeding 0.9812. This indicates that FCLS has high accuracy and stability when dealing with the complex mixed spectra of biological samples. This provides a reliable technical foundation for future medical HSI applications, particularly for the spectral unmixing of biological or diseased tissues.

Overall, this study successfully detected targets at different depths in collagen phantoms and accurately estimated the collagen concentrations. This not only verifies the feasibility of SWIR HSI in biomedical applications but also demonstrates the usefulness of phantom experiments for validating algorithms and conducting reproducible research. Furthermore, this study provides theoretical and technical support for the development of more precise noninvasive diagnostic tools.

However, this study has some limitations. First, as the detection depth increased, the accuracy of the image analysis decreased, likely owing to light scattering and absorption in deeper tissues. Second, although the collagen phantom model used in this study simulated biological tissue properties, it lacked the complexity of real *in vivo* tissues. Future studies should focus on the following areas: First, the spectral imaging technology is optimized to reduce the effects of light scattering and absorption in deeper tissues, which may involve improving the light source design or using more advanced image-processing algorithms. Second, this technology can be applied to more complex *in vivo* tissue models to verify its feasibility and reliability in clinical settings. Finally, we must explore whether this imaging technique can be combined with other diagnostic tools such as multimodal imaging systems to enhance its clinical application and diagnostic accuracy.

## 5 Conclusion

This study tested the ability of SWIR HSI to penetrate collagen phantoms of different thicknesses. The results show that this technology has high potential for biomedical applications. Our experiments proved that even with a collagen phantom thickness of 60 mm, the proposed algorithm could effectively detect the targets. This shows that SWIR light has a strong penetration power in collagen phantoms, which can be used in future studies to design and validate hyperspectral image-processing algorithms.

When the CEM method was used to detect subpixel targets (lards) in a collagen phantom, the lards were accurately detected at depths of 7 to 20 mm. However, when the lard was embedded deeper than 20 mm, CEM detection accuracy decreased, indicating that light scattering and absorption significantly affected the detection of deeper targets.

This study also used the FCLS method to perform a spectral unmixing analysis of collagen phantoms with different concentrations and lard–collagen phantoms with slope distributions. The results showed that the FCLS method accurately estimated collagen concentrations of 2% to 5% and accurately estimated the lipid (lard) distribution fractions at different depths. The correlation coefficient between the estimated results and the GT values reached 0.9917, whereas Spearman’s correlation between the estimated lard distribution fractions and the slope depths exceeded 0.9812, indicating that LSU performed well in analyzing complex sample spectra.

This study successfully used different collagen phantom experiments to verify the potential application of SWIR HSI and related analytical algorithms in biomedicine. It provides reliable technical support for the development of non-invasive diagnostic tools. Future research should focus on validating other image processing and analysis techniques, exploring the effects of light scattering in deep tissues, and developing more complex living tissue models to further optimize and validate these technologies for clinical applications.

## Data Availability

The data supporting the findings of this study are available from the corresponding author upon request.
